# Microbiological Assessment, Nutritional Characterization and Phenolic Compounds of Bee Pollen from *Mellipona mandacaia* Smith, 1983

**DOI:** 10.3390/molecules200712525

**Published:** 2015-07-09

**Authors:** Marivalda Santa Bárbara, Cerilene Santiago Machado, Geni da Silva Sodré, Luís G. Dias, Leticia M. Estevinho, Carlos Alfredo Lopes de Carvalho

**Affiliations:** 1 Grupo de Pesquisa Insecta, Centro de Ciências Agrárias, Ambientais e Biológicas da Universidade Federal do Recôncavo da Bahia, 44380-000 Cruz das Almas, Bahia, Brazil; E-Mails: marivaldafsb@gmail.com (M.S.B.), cerilenes@yahoo.ufrb.edu.br (C.S.M.); geni@ufrb.edu.br (G.S.S.); calfredo@ufrb.edu.br (C.A.L.C.); 2 Escola Superior Agrária, Instituto Politécnico de Bragança, Campus Santa Apolónia, 5301-855 Bragança, Portugal; E-Mail: ldias@ipb.pt; 3 Centro de Química-Vila Real, Universidade de Trás-os-Montes e Alto Douro, 5000-801 Vila Real, Portugal; 4 Centro de Investigação de Montanha, Escola Superior Agrária, Instituto Politécnico de Bragança, Campus Santa Apolónia, 5301-855 Bragança, Portugal

**Keywords:** bee pollen, fatty acids, *Melipona mandacaia*, microbiological quality, nutritional characterization, stingless bee

## Abstract

This study aims to assess the microbiological parameters and the chemical composition of 21 samples of stingless bee pollen (*Melipona mandacaia*) from two regions of Bahia, Brazil (João Dourado and Uibaí), with particular emphasis on the nutritional value, total phenols and flavonoids and fatty acids composition. Regarding the microbiological quality, the studied microorganisms (moulds and yeasts, coliforms, *Escherichia coli*, *Staphylococcus aureus*, *Salmonella* sp., psychrotrophic and sulfite-reducing Clostridia) were absent in all samples. On the other hand, the values obtained for the aerobic mesophilic microorganism ranged from 11.0 ± 1.0 to 1.32 ± 1.2 cfu∙g^−1^ (JD samples) and from 282 ± 3.82 to 688 ± 10.1 cfu∙g^−1^ (U samples). The nutritional parameters (moisture, ash, water activity, pH, total acidity, protein, fiber, total phenolic, flavonoids and reducing sugars) were within the stipulated by law, except for pH and moisture content, which presented superior and inferior values, respectively. Polyunsaturated fatty acids (54.1%) were significantly higher than saturated (42.18%) and monounsaturated (3.71%). It was found that the bee pollen is safe from the microbiological point of view and has a good nutritional quality. The influence of the geographical origin on the assessed parameters was evident, especially concerning the fatty acid profile.

## 1. Introduction

For several years, meliponiculture was considered a recreational activity, since colonies were thought to be low productive when compared with the ones from the honey bee *Apis mellifera* [1]. However, recent evidence supports that those easily reared bees constitute a feasible and important source of income for beekeepers, providing considerable amounts of high-quality products [2].

In Brazil, *Melipona* bees are indigenous stingless bees and present all over the country, although species differ according to the region [3]. *Melipona mandacaia* (*Mandacaia* stingless bee) is widespread in the Brazilian states of Bahia, Ceará, Paraíba, Pernambuco and Piauí, usually occurring around the São Francisco and the Vaza Barris rivers [4]. Besides their importance as major pollinators of most wild plants and some cultivated species, the products obtained from this species are also useful sources of therapeutically active compounds and play an important role in the nutritional industry [5].

Indeed, it has been claimed that the products obtained from meliponiculture possess distinctive characteristics when compared to the produced by *Apis mellifera*, particularly concerning the sensory properties, receiving higher ratings and meeting the demands of the consumers [6]. However, little evidence is available on the literature regarding the composition of these products, what hampers their further uses in the food and pharmaceutical industry.

Among these products, special emphasis may be laid on bee pollen, a natural product composed by flower pollen that bees collect and mix with their own specific substances, forming the pellets that are then placed in the honeycomb cells [7]. This beehive product has been used traditionally for human consumption, as well as in folk and complementary medicine. Indeed, recent evidence reports valuable biological properties, among them: antimicrobial, anti-inflammatory, antimutagenic [8], antioxidant [7], anti-allergic [9], antiviral, hypolipidemic, hypoglycemic and immunostimulating [10].

Bee pollen is rich in carbohydrates, crude fibers, lipids, vitamins and phenolic compounds, being commonly named as the “perfectly complete food”, due to the presence of all the essential amino acids [11]. In spite of this, the specific chemical composition of this natural product depends strongly on the bees’ specie, botanical and geographic origin, as well as on other factors, among which the climatic conditions, soil type and agricultural practices [8].

This study aims to assess the microbiological parameters and to report the chemical composition of 21 samples of bee pollen from *Mandacaia* stingless bee (*Melipona mandacaia*) from two regions of Bahia (João Dourado e Uibaí) in Brazil, with special interest in the nutritional value, total phenols and flavonoids and fatty acids composition. As far as the authors know, there is no literature regarding bee pollen from this bee specie, despite the increasingly higher scientific and socio-economic importance of this product.

## 2. Results and Discussion

A total of 21 samples of bee pollen collected from two regions of Bahia (João Dourado and Uibaí) in Brazil, were analyzed in triplicate by microbiological assays and chemical evaluation.

Due to the absence of literature specifications for the pollen collected by the *Melipona* bees, the pollen from *Apis mellifera* was used as element of comparison, based on the legislation of different countries and works done by various researchers [8].

### 2.1. Microbiological Contamination

Regarding the results obtained in the microbiological analyses, it was noted that the *Salmonella* sp., sulfite-reducing clostridia, fecal coliforms, *Escherichia coli*, *Staphylococcus aureus*, yeast and moulds were absent in all samples. These results showed that, microbiologically, the analyzed pollen samples were safe and therefore able to be used as a food product. These analyses were performed since microbial contaminations are possible, mainly associated with the pollens’ processing, as observed in other works, for example, Coronel *et al.* [12] detected contaminations by faecal coliforms in twenty-three bee pollen samples and Estevinho, *et al.* [13] detected yeast and moulds in 60% of pollen samples, though in lower concentration. In Table 1 it are presented the determinations for the mesophilic aerobic microorganisms.

The mesophilic aerobic microorganisms were present in all the analyzed pollen samples and with high variability in concentrations (85 ± 63 cfu∙g^−1^ for JD and 443 ± 142 cfu∙g^−1^ in U). All the results were within the limits established by Argentine Food Code [14], whose maximum value was set at 1.5 × 10^3^ cfu∙g^−1^ for aerobic mesophilic microorganisms at 30 °C. This suggests that the hygiene conditions during harvest, cleaning, drying and storage were adequate.

**Figure 1:**
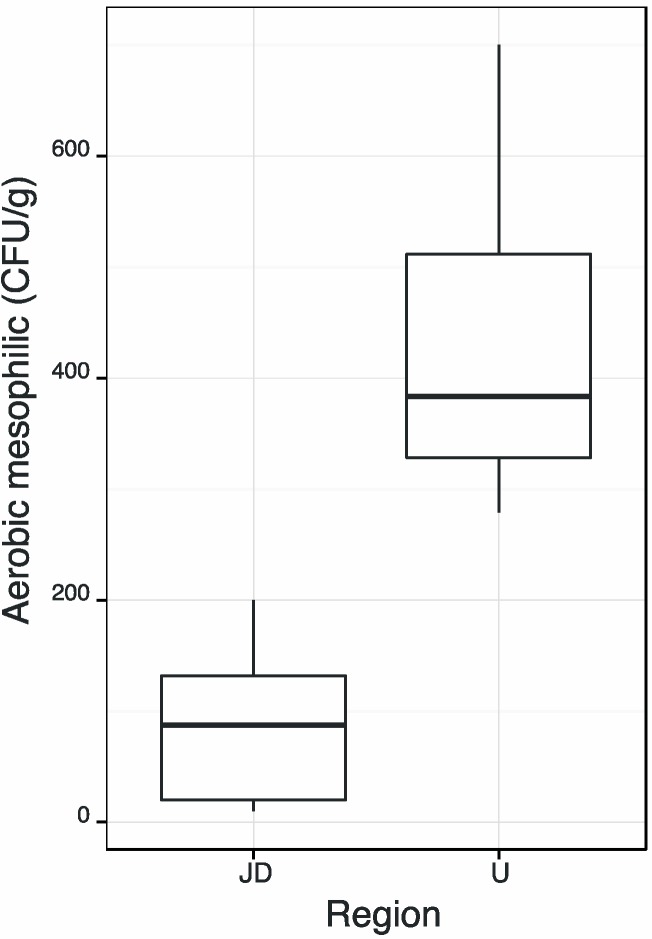
Boxplot for aerobic mesophilic bacteria.

In order to evaluate which statistical test could be used to verify if mesophilic aerobic microorganisms amounts present in samples were influenced by the pollens’ origin (region João Dourado, JD, and Uibaí, U), it was checked whether the data presented normality and homogeneity of variances. For each group, the Shapiro-Wilk test showed that the data had no normal distribution (*p* < 0.006 at α = 0.05) and the Levene’s test that between the two-formed groups there was no homogeneity of variance (*p* < 0.001 at α = 0.05). Considering this situation, the Welch test was applied for checking equality between groups’ averages. This test showed that the averages of obtained values for mesophilic aerobic microorganisms in both regions had not significant differences (*p* < 0.001 at α = 0.05). The Figure 1, shows the boxplot chart for mesophilic aerobic microorganisms of each region that corroborates with previous findings because, it was possible to show that the amounts of aerobic mesophilic microorganisms were higher in samples from U region (with an average 5.2 times higher than those presented in samples from JD region) and also with greater variability.

**Table 1:** Results obtained for the aerobic mesophiles (cfu∙g^−1^) in the bee pollen samples from the two locations.

João Dourado																			
Sample	JD1	JD2	JD3		JD4		JD5		JD6		JD7		JD8		JD9		JD10		JD11
Aerobic mesophiles (cfu∙g^−1^)	66 ± 3.6	19.7 ± 1.5	132 ± 1.2		10.9 ± 1		195.64 ± 4.56		21.33 ± 1.53		87.80 ± 2.97		11.00 ± 1.00		162.11 ± 2.64		11.17 ± 1.26		121.58 ± 1.41
Uibaí																			
Sample	U1	U2		U3		U4		U5		U6		U7		U8		U9		U10	
Aerobic mesophiles (cfu∙g^−1^)	514.21 ± 8.51	497.48 ± 2.32		678.27 ± 3.41		307.20 ± 2.06		350.39 ± 58.88		385.60 ± 4.13		688.40 ± 10.07		352.83 ± 2.46		282.15 ± 3.82		375.67 ± 5.30	

### 2.2. Nutritional Characterization and Phenolic Compounds

The physical and chemical parameters analyzed were: moisture content, ash content, water activity (a_w_), pH value, total acidity, protein content, fiber content, total phenolic content, flavonoid content and reducing sugar content. The results showed that half of these parameters had values varying in a small interval, with standard deviation values percentage ranging between 1.10% and 6.60%. These parameters were the moisture content, which had an average value of 36.0 ± 2.0%; the ashes, with a percentage of 4.9 ± 0.3%; and, the water activity with 0.86 ± 0.02% (values obtained at 26.7 ± 0.8 °C). In addition, the average values of pH were 3.49 ± 0.04 and total acidity, 146 ± 10 meq∙kg^−1^. Regarding the other parameters under study, the relative standard deviation percentages ranged from 11% to 39%: protein content presented an average value of 21 ± 2%; average fiber content was 3.6 ± 1.4%; total phenolic content was 40 ± 13 mg GAE/g; flavonoid content was 1.0 ± 0.2 mg CAE/g; and, total sugars content was 12 ± 2%.

The obtained values for pH are lower than those established by Brazilian legislation [15] and Argentine Food Code [14], as well as, obtained by Coronel *et al*. [12] and Marchini *et al*. [16], in dehydrated pollen samples from Argentina and Brazil, respectively. With regard to *a*_w_, ash and moisture analyses, the obtained values were higher than those found by Morais *et al*. [7], Feás *et al*. [11] and Nogueira *et al*. [17] in dehydrated pollen samples from Portugal. The ash content was in accordance with the Argentine Food Code’s established limits [14], *i.e.*, between 2% and 5%, and also with those obtained by Almeida-Muradian *et al*. [18] (2.1 ± 0.8%) and Bonvehi *et al*. [19] (4.8%) in dehydrated pollen samples from Brazil and Spain, respectively. All the samples showed high moisture content, exceeding the maximum established limits by Brazil’s legislation (30% in bee pollen and 4% in dehydrated pollen), Argentina (8% in bee pollen), Bulgaria (10%), Poland and Switzerland (6%) [20]. According to Marchini *et al*. [16] this may be related to the fact that pollen, a hygroscopic product, is easily affected by environmental conditions. The protein content in pollen was similar to those obtained by Almeida-Muradian *et al*. [18], González-Martín *et al*. [21] and Carpes [22] in pollen samples from Brazil and Spain. Estevinho *et al*. [13] presented results of Portuguese pollen protein between 24% and 34%, values slightly higher than those found in this study. The content of reducing sugars was much lower than the values obtained by Carpes [22] (49 ± 4%) in pollen samples from Brazil and by González-Martín *et al*. [21] in Spanish pollen samples (23% to 41%). According to Silva *et al*. [23] and Carpes *et al*. [24], the reducing sugar content in pollen is associated with the amount of honey or nectar present in the product. The fiber content and the free acidity are within the limits stipulated by Brazilian law (2% minimum of fibers and 300 meq∙kg^−1^ maximum of free acidity) [15].

The concentrations of phenolic compounds obtained in the present study were slightly higher than the reported by Carpes *et al*. in *Apis mellifera* bee pollen samples from Brazil [25] and by Morais *et al.* [7] and Feás *et al.* [11], who studied *Apis mellifera* bee pollen from Portugal. However, the results of other studies are within the range of the hereby reported, for example, in *Apis mellifera* samples from Southern Brazil [22], in pollen pellets from the USA [26] and in different Iberian commercial bee pollens [17]. In this context, even though the differences obtained are not very relevant, these may be assigned, mainly, to differences in the bee species but also to the diverse botanical and geographical origins of the studied products [25,27].

Regarding to the content of total flavonoids, the pollen samples analyzed showed lower values than those found by LeBlanc *et al*. [26], Mărghitas *et al*. [28], Feás *et al*. [11] and Carpes [22], pollen samples from North America, Romania and Brazil, respectively.

In the collinearity study, using all variables, the linear relationships presented correlation coefficients (R) lower than 0.5, with the exception of 2 relationships: moisture content *versus* ash content (R = −0.67 with *p* < 0.001); total phenolic *versus* flavonoid content (R = 0.63 with *p* < 0.001).

**Figure 2:**
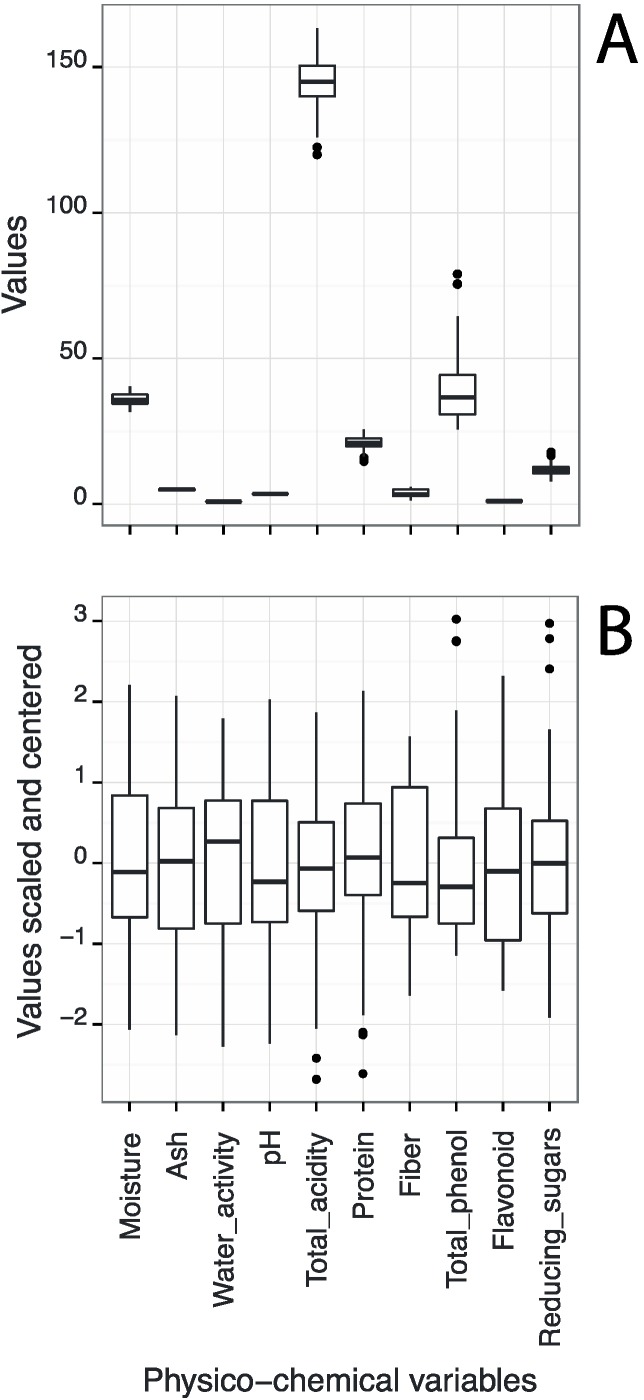
Two boxplot graphs of all physical and chemical parameters measured in the samples: (**A**) raw data; (**B**) centralized and standardized data.

These results are in agreement with the fact that the range of variable values was not very large in order to obtain any relevant linear tendency. Figure 2 presents two boxplot graphs of all physical and chemical parameters measured in the samples: (A) raw data; (B) centralized and standardized data. Figure 2A allows visualizing the variability of variables into a single scale and to identify the extreme values.

Firstly, it was possible to check that the variables had different order of magnitude, indicating that when using multiple variables, they should be centralized and standardized so that all have the same importance in the statistical study. Secondly, the extreme values (better visualized in the Figure 2B) identified in the boxplot graph were: total acidity in U4 samples, protein content in J10 samples, total phenolic content in the U10 samples and total sugar content in JD7 samples. These extreme values were not common to other variables and so, the respective samples could not be considered as outliers.

**Figure 3:**
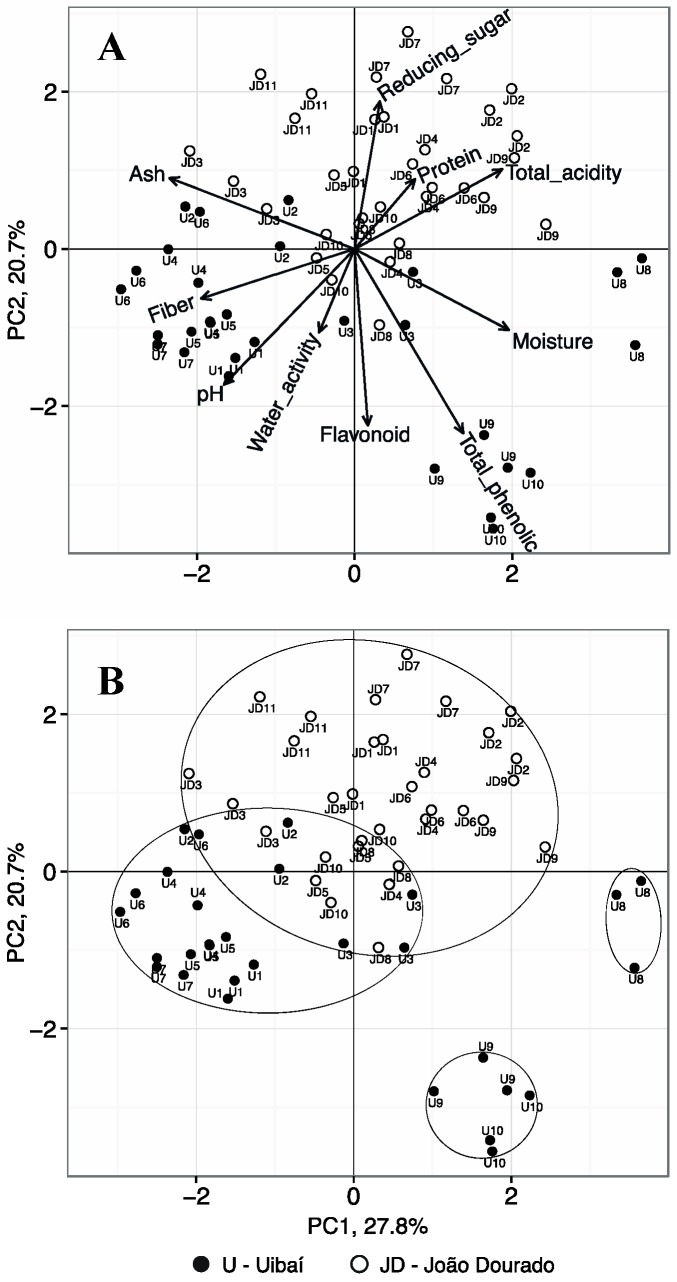
Representation of the first two principal components for all pollen samples distinguishing the place of origin by using different markers: plot (**A**) includes loadings of the original variables; plot (**B**) includes possible outline grouping samples.

To verify samples similarity considering these physical and chemical characteristics, principal component analysis, an unsupervised multivariate method for exploratory analysis, was applied. The method gave 10 principal components (PCs) that explained 100% of overall data variance; PC1 explained 28% of the total variance, while PC2 explained 21%. The following three PCs represent 15%, 10% and 9% of the total data variance, while the remaining PCs had values between 0.8% and 6%. Figure 3 shows two graphs of the representation of samples scores in the first two principal components (PC1 and PC2), emphasizing the place of origin of each sample by using different markers; in the first plot (A) it is shown the loadings of the original variables; in the second plot (B), possible outline grouping samples is proposed. Other representations with other PC functions have been considered but it was verified that they showed no relevant information for the study. Figure 3 allowed to visualize that most samples from JD region are separated from the ones from U region, showing that the origin of the sample contributes to the physical and chemical characteristics.

Samples from JD region, in general, are characterized by having higher values of total acidity, protein content and total sugar content and low/intermediate values of water activity, pH values, fiber content, flavonoid content and total phenolic content. Regarding the U samples, most had the opposite tendency, with the exception of samples U8, U9 and U10, which deviated from all others by presenting higher levels of total phenolic compounds and moisture content.

Prior to assessing the significance of the differences between the results of the two locations, it was evaluated the normality of the data using the Shapiro-Wilk test. The variables total acidity, protein, fiber, total flavonoids and reducing sugars showed lack of normality (*p* < 0.033) for JD pollen samples, while for U pollen samples the same behavior was observed for pH, fiber and total flavonoids (*p* < 0.012) (instead of total acidity, protein content, fiber content, flavonoid content and the total sugar content of JD samples showed lack of normality (*p* < 0.033); the variables of U samples that showed lack of normality were the pH values, fiber content and flavonoid content (*p* < 0.012).

To confirm these results the normal QQ plots were obtained. It was possible to verify that a low degree of skew in lower values was present in distribution of same variables but all can be (instead of was present in some variable distribution but not all can be.

Secondly, the Levene’s test was used to assess the homogeneity of variances. Moisture, total acidity, fiber and reducing sugars parameter showed variance homogeneity (*p* > 0.57) in the data of two regions investigated (instead of Moisture content, total acidity, fiber content and total sugar content had variances homogeneity (*p* > 0.57) in the data of both regions. Anova one-way was applied and significant differences were found between the results obtained for the two regions for all the parameters, except moisture content (*p* = 0.78).

Regarding the variables ash content, water activity, pH values, protein content, total phenolic content and flavonoid content here was no homogeneity of variances. In this case, a Welch Anova was applied. The variables ash content, pH values, water activity and protein content did not presented significant differences between samples of each region (*p* > 0.070). In contrast, the total phenolic and flavonoid content differed significantly (*p* < 0.003). Globally, the variables total acidity, fiber, total phenolic, flavonoid and total sugar contents, had relevant information that allow to distinguish samples from the two regions and their contribution towards samples distinction is evident in Figure 3.

### 2.3. Fatty Acid Profile

Overall, it was possible to identify 15 different fatty acids present in the samples by gas chromatography analysis, being 9 saturated and 6 unsaturated fatty acids: caprylic acid (C8:0), capric acid (C10:0), lauric acid (C12:0), myristic acid (C14:0), pentadecylic acid (C15:0), palmitic acid (C16:0), margaric acid (C17:0), stearic acid (C18:0), oleic acid (C18:1n9ct), linoleic acid (C18:2n6c), α-linolenic acid (C18:3n3), arachidic acid (C20:0), eicosatrienoic acid (C20:3n3), erucic acid (C22:1n9) and nervonic acid (C24:1). These fatty acids were already identified in pollen samples studied in other works [29,30], except the erucic and nervonic acids that, according to information gathered by the authors, is the first time it is mentioned. Some of the identified fatty acids were not detected in all pollen samples. Four fatty acids (C8:0, C12:0, C20:3n3 and C24:1) were not detected in more than 5 samples, present in both regions, while the C22:1n9 fatty acid was not detected in 2 samples of the JD region and the C17:0, in 2 samples of U region. It should be noted that, in samples of JD region, the fatty acid C20:3n3 had levels generally lower or was not detected in a larger number of samples than samples of the U region. Table 2 shows the mean and standard deviation values of the overall fatty acids results as well, partial results, for each of the regions under study. The chromatographic profile obtained show that the palmitic acid, linoleic acid and linolenic acid (C16:0, C18:2n6c and C18:3n3, respectively) were those with mean amounts higher than > 18% of total fatty acids. But, while the two regions had similar mean levels of the palmitic acid, pollens from JD region had higher mean levels of linoleic acid (twice the amount obtained in pollens of the U region) and, on the contrary, the linolenic acid had low levels, almost half of those found in samples of the U region. The remaining fatty acids showed mean levels lower than 2.3% and 4.4% in pollens of JD and U regions, respectively. Moreover, standard deviation values showed a high variability within the results for these fatty acids with low levels, which shows that, in this work, there was an intrinsic variability in samples used, even the ones from the same region.

Globally, the levels of unsaturated fatty acids were generally higher than those of saturated fatty acids, presenting levels between 43% and 70% of the total fatty acids. Also, in general, polyunsaturated fatty acids were significantly higher than monounsaturated and total saturated fatty acids. The ratio of unsaturated/saturated fatty acids was 1.4 ± 0.4. Only 4 samples showed contrary tendencies, although in two samples the results between repetitions were not concordant. This ratio tendency was already reported in a previous work [13], which states that these results are in accordance to the hypothesis that the bees choose pollens with higher content of unsaturated fatty acids (more adequate to their metabolism). From a nutritional point of view, this ratio shows that this pollen can be an asset if added to processed food by improving the nutritional quality of the manufactured food [13].

**Table 2:** Descriptive analysis of pollen’s fatty acids.

Fatty Acid Common Name	Fatty Acids	All Samples (%)		Samples of JD Region (%)		Samples of U Region (%)	
		Mean	SD	Mean	SD	Mean	SD
Caprylic acid	C8:0	0.11	0.11	0.14	0.13	0.09	0.08
Capric acid	C10:0	0.28	0.17	0.42	0.05	0.12	0.11
Lauric acid	C12:0	0.06	0.04	0.05	0.04	0.06	0.02
Myristic acid	C14:0	0.28	0.11	0.32	0.12	0.24	0.08
Pentadecylic acid	C15:0	1.28	1.10	2.00	1.04	0.50	0.41
Palmitic acid	C16:0	34.99	4.88	34.10	5.28	35.96	4.33
Margaric acid	C17:0	0.36	0.27	0.54	0.25	0.17	0.10
Stearic acid	C18:0	1.97	0.88	1.36	0.51	2.65	0.67
Oleic acid	C18:1n9ct	1.79	1.00	2.22	0.84	1.33	0.99
Linoleic acid	C18:2n6c	27.79	9.91	36.58	4.04	18.12	2.51
α-Linolenic acid	C18:3n3	26.08	10.03	18.85	6.47	34.03	6.62
Arachidic acid	C20:0	2.85	2.02	1.44	0.85	4.40	1.77
Eicosatrienoic acid	C20:3n3	0.23	0.25	0.14	0.24	0.33	0.22
Erucic acid	C22:1n9	1.12	0.53	0.87	0.48	1.40	0.44
Nervonic acid	C24:1	0.80	0.50	0.97	0.48	0.61	0.45

In Table 3 is shown the correlation matrix obtained by testing linear dependence between pairs of variables (fatty acids) using Pearson’s correlation coefficients. The table shows that only 10 relations between pairs of variables had reasonable linear trend (positive and negative slopes), with Pearson correlation coefficient values between 0.71 and 0.87; positive correlation coefficients and greater than 0.7 were obtained between pairs of variables C10:0–C18:2n6c (R = 0.87), C15:0–C17:0 (R = 0.71), C17:0–C18:2n6c (R = 0.74) and C18:0–C20:0 (R = 0.84); those with negative values, and absolute values higher than 0.7 were found for the relations C10:0–C18:3n3 (R = −0.80), C10:0–C20:0 (R = −0.82), C18:0–C18:2n6c (R = −0.74), C18:2n6c–C18:3n3 (R = −0.79), C18:2n6c–C20:0 (R = −0.73) and C18:3n3–C24:1 (R = −0.71). No linear relation with absolute values of correlation coefficients higher than 0.9 were found and, so, all the fatty acids were considered in the following studies.

**Table 3:** Correlation matrix for the fatty acids using Pearson’s correlation coefficients.

R-Pearson Values	C8:0	C10:0	C12:0	C14:0	C15:0	C16:0	C17:0	C18:0	C18:1n9ct	C18:2n6c	C18:3n3	C20:0	C20:3n3	C22:1n9	C24:1
C8:0	1														
C10:0	0.1	1													
C12:0	−0.29	0	1												
C14:0	0.27	0.43	0.13	1											
C15:0	0.27	0.66	−0.32	0.53	1										
C16:0	0.42	0	−0.17	0.26	0.05	1									
C17:0	0.19	0.66	−0.11	0.11	0.71	−0.15	1								
C18:0	0.15	−0.67	0.03	−0.15	−0.56	0.54	−0.63	1							
C18:1n9ct	−0.08	0.49	0.40	0.37	0.25	−0.43	0.29	−0.45	1						
C18:2n6c	0.14	0.87	−0.05	0.32	0.65	−0.26	0.74	−0.74	0.48	1					
C18:3n3	−0.41	−0.80	0.11	−0.52	−0.67	−0.35	−0.62	0.31	−0.24	−0.79	1				
C20:0	0.1	−0.82	−0.09	−0.27	−0.48	0.32	−0.59	0.84	−0.60	−0.73	0.43	1			
C20:3n3	−0.43	−0.17	0.21	0.01	−0.17	0.08	−0.03	0.19	0	−0.28	0.19	0.06	1		
C22:1n9	−0.19	−0.44	0.19	−0.07	−0.55	0.42	−0.53	0.68	−0.38	−0.53	0.20	0.51	0.42	1	
C24:1	0.24	0.55	0.27	0.48	0.22	0.51	0.22	0.05	0.22	0.38	−0.71	−0.27	0.09	0.24	1

**Figure 4:**
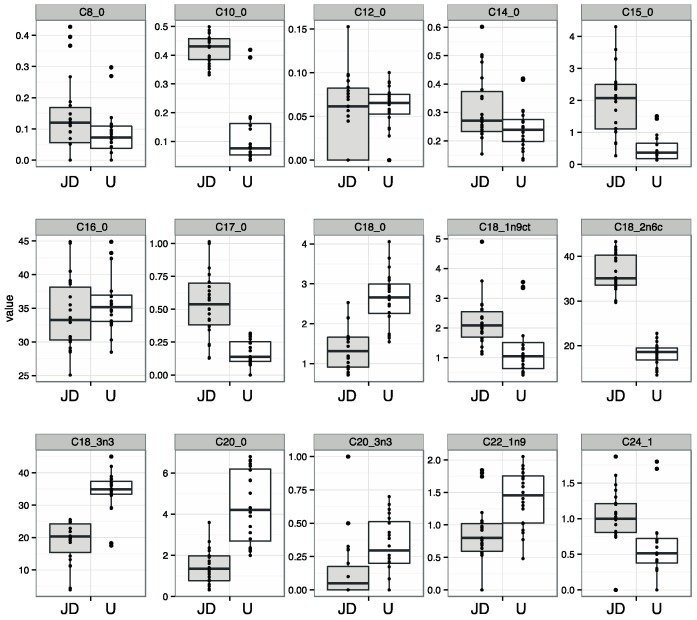
Box-plot for each fatty acid grouped according to sample’s region.

In Figure 4, boxplots for each fatty acid are presented allowing to visualize the data obtained for the pollen samples of the two regions and the variability found within each group. The figure showed plots with extreme values considered typical results (not outliers) since the samples did not showed the same trend in other variables.

Through the Shapiro-Wilk normality test, it was shown that the fatty acids variables without normality (*p* < 0.007) were: C8:0, C10:0, C12:0, C14:0, C15:0, C17:0, C18:1n9ct, C18:2n6c, C20:0 e C20:3n3. With p-values higher than 0.09, the variables presenting normality were C16:0, C18:0, C18:3n3, C22:1n9 e C24:1.

To assess the homogeneity of variances in each fatty acids, considering the groups defined, it was applied the Levene test. The variables that had variance homogeneity (*p* > 0.05) were: C8:0, C10:0, C14:0, C16:0, C18:0, C18:1n9ct; C18:3n3, C20:3n3, C22:1n9 and C24:1. For the variables that meet the assumptions of normality and homogeneity of variances, it was applied the Anova one-way to verify if there were significant differences between the mean values of samples from the two regions. Also in this scope, for those non-normal variables, non-parametric methods were used; the Kruskal-Wallis test, if variables were homoscedastic, or Welch test (Anova with Welch’s correction), if the variables were heteroscedastic.

The Anova method showed that only the fatty acid C16:0 had no significant differences between the two groups of samples (*p* = 0.22). With the non-parametric tests, it was found that only the fatty acids C8:0 and C12:0 presents no significant differences (*p* > 0.12) while the others variables showed significant differences among the samples of the two regions (*p* < 0.036). The 3 variables with no significant differences between groups of samples (C8:0; C12:0 and C16:0) were not used in the PCA study. The remaining variables, by having results with different magnitudes, were centered and scaled. Therefore, the data matrix for PCA was constructed using the results of 12 fatty acids analyzed in the 21 pollen samples.

**Figure 5:**
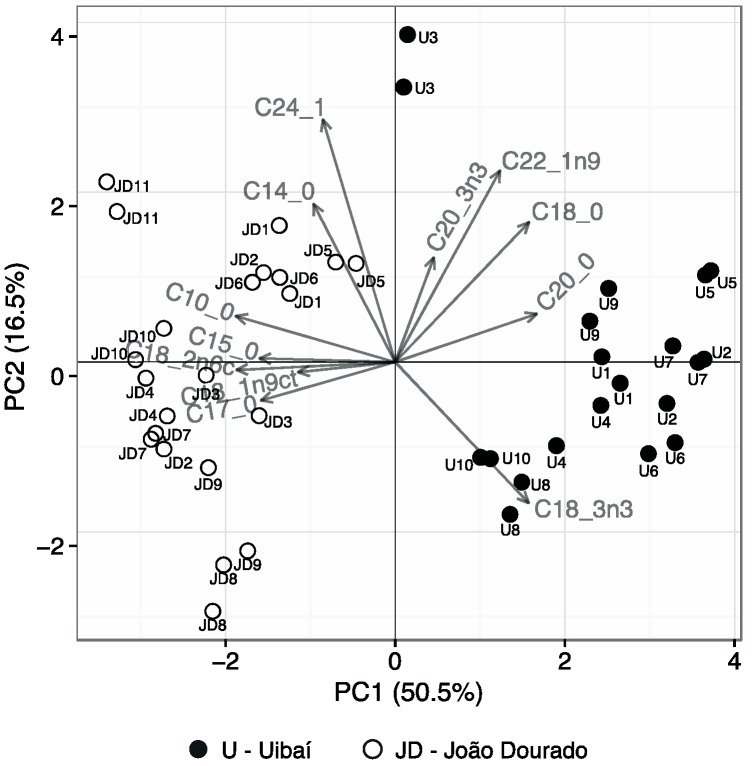
The first two principal component scores of the pollen’s fatty acids.

The results of PCA showed that 7 principal components were sufficient to explain 96% of the total variance of the data. The first and second principal components explained 67% of the data’s total variance. Figure 5 shows the representation of the first two principal component scores obtained for the results of the fatty acids analyzes.

It was possible to visualize the behavior of the samples variability, showing that pollen samples could be separated in two groups, according to the pollen’s place of origin. Using the first principal component (PC1), it was possible to separate the pollen samples of JD region, present in the negative quadrant, from the U region, present in the positive quadrant. The figure shows that the samples of U region were grouped in PC1 due mainly to high levels of C18:0, C18:3n3, C20:0, C20:3n3 and C22:1n9 fatty acids. Only the U3 sample deviates from this grouping by having overall median levels of fatty acids in the PC1 dimension but, mainly because it had higher values in the second principal component (PC2). Otherwise, in the same dimension, pollen from JD region showed to have a lipid profile with higher levels in the fatty acids: C10:0, C14:0, C15:0, C17:0, C18:1n9ct, C18:2n6c and C24:1. In this group, the sample JD8 also showed to have a distinctive profile over all other JD samples, which is emphasized by the PC2.

## 3. Experimental Section

### 3.1. Pollen Samples

In this study, 21 samples of bee pollen were collected in the honeycombs of *Melipona mandacaia* (Mandacaia bee) in two regions: 11 samples from João Dourado (JD: 11°21′00′′ S 41°39′50′′ O) and 10 samples from Uibaí (U: 11°20′13′′ S 42°07′58′′ O), two municipalities belonging to the micro-region Irecê, in the state of Bahia in the North-East region of Brazil. These two regions have differences in their altitudes reflected in the diversity of flora. Environmental conditions found in Uibaí allow higher humidity, resulting in higher plant diversity. In this environment, the native vegetation is still preserved. In contrast, the region of João Dourado has lower humidity and greater human influence, especially in agro-pastoral activities, reflected in a lower diversity of native species. These differences in floristic composition between the two regions can influence the physical and chemical composition of pollen stored by *Mellipona mandacaia* Smith bee. Each sample was identified using letters—U or JD, representing the geographic origin, and numbers. The preparation of the extracts was performed as described by Morais *et al.* [7], by mixing the bee pollen with methanol (1:2) (*w*/*v*). After maceration, the bee pollen extract was evaporated in a vacuum evaporator and the dried extract was kept in the dark at room temperature. All the analyses were carried out in triplicate, yielding 63 results for each parameter.

### 3.2. Microbiological Determinations

In order to evaluate the microbial quality of the *Melipona mandacaia* bee pollen samples, they were analyzed for the presence of mesophilic microorganisms, yeasts and moulds, fecal coliforms, *Escherichia coli*, *Staphylococcus aureus*, sulphite reducing clostridium spores and *Salmonella*.

#### 3.2.1. Sample Preparation

A mass of 25 g of bee pollen were aseptically taken and homogenized for 3 min with 225 mL of pre-chilled (4 ± 0.5 °C) sterile peptone-physiological saline solution (0.1% neutral peptone + 0.85% NaCl in sterile deionized L1O, pH = 7.0 ± 0.05). Decimal serial dilutions were prepared from this homogenate in the same chilled sterile diluents (1:10 *v*/*v*) [31].

#### 3.2.2. Enumeration of the Total Mesophilic Microorganisms

The total quantification of aerobic mesophilic microorganisms was done following the Portuguese regulations [32], with sowing by incorporation of 1 mL for each decimal dilution to Petri plates culture medium containing Plate Count Agar (PCA-Himedia, Mumbai, India). The colony counting was made after incubation plates at 30 °C for 72 h and the results were expressed as colony forming units per gram of bee pollen (cfu∙g^−1^).

#### 3.2.3. Enumeration of Yeast and Moulds

Moulds and yeasts enumeration was made on DG18 and incubated at 25 °C for 5 days. Microbial counts were expressed as cfu∙g^−1^ [33].

#### 3.2.4. Sulphite Reducing Clostridium Spores

The quantification of clostridium sulphite-reducing spores was performed according to the ISO norm [34]. First, 1, 5 and 10 mL of the stock suspension were placed in the test tube. The tubes were heated in a water bath at 80 °C for 10 min. Subsequently, the samples were split into Petri dishes containing the iron Sulphite agar base (Himedia). Then, it was added another layer of agar base in the middle plate and sealed with parafilm. After solidification, the plates were incubated at 37 °C for 72 h. The appearance of black colonies on the plates was considered as a positive result. Results were expressed as most probable numbers of sulphite-reducing Clostridium spores per gram of bee pollen (MPN∙g^−1^).

#### 3.2.5. Enumeration of Fecal Coliforms and *Escherichia coli*

For the quantification of total coliforms and *Escherichia coli*, SimPlate kit system from Bio Control, was used. The supplied culture medium was pre-hydrated in 9 mL of distilled water, inoculated with 1 mL of the stock suspension and homogenized by vortex, following the manufacturer’s recommendations. The contents were poured onto plates containing 84 wells. The liquid was evenly spread across the wells in a circular motion at low speed and the excess was removed. The plates were incubated at 35 °C for 24 to 48 h. The enumeration of total coliforms was performed by counting the number of wells in which the color change occurred in the culture medium. The enumeration of *E. coli* was performed by counting the number of wells in which the fluorescence was observed after exposure to a plate of ultraviolet light (UV) at 365 nm. The number of coliforms present in the sample was calculated according to the table provided by the manufacturer [35].

#### 3.2.6. Detection of *Salmonella* sp.

The detection of *Salmonella* sp. was carried out using the immunodifusion 1–2 test, as described in the AOAC method [36]. The results were obtained 16–20 h after pre-enrichment in buffered peptone water and the results were interpreted visually, by monitoring the development of an immunoband, which is a characteristic immobilization pattern of cells.

#### 3.2.7. Determination of *Staphylococcus aureus*

Serial dilutions of the sample were inoculated in Baird-Parker Broth with Egg Yolk Tellurite and Sulfadimidine Solution (Himedia) during 24 h (37 °C). Then, three to five characteristic colonies were selected, in order to verify the presence of coagulase and catalase. Microbial counts were expressed as colony-forming units per gram of bee pollen (cfu∙g^−1^) [37].

### 3.3. Physicochemical Analyses

#### 3.3.1. Moisture Content

Approximately 2 g of each sample of the *Melipona mandacaia* bee pollen was dried at a temperature of 105 °C, till constant weight [38].

#### 3.3.2. Ash Content

The ash content was determined after ignition at 550 ± 15 °C by gravimetry [39].

#### 3.3.3. Water Activity

This parameter was determined by placing each pollen sample directly into a water activity meter (Rotronic HygroPalm. Bassersdorf CH-8303) [40].

#### 3.3.4. pH Values

pH was measured in the aqueous phase obtained after mixing 10 g of pollen in 75 mL of distilled water [40] by a pH combined electrode connected to a pH 211 Microprocessor pH Meter of Hanna Instruments.

#### 3.3.5. Total Acidity

Regarding total acidity, 2.5 g of bee pollen were mixed with CO_2_-free water and the titration was started with NaOH 0.05 N in a 5 mL per minute flow, until reaching the pH 8.5. This determination was carried out according to AOAC norm [40].

#### 3.3.6. Protein Content

Nitrogen content was determined using the Kjeldahl method (230-Hjeltec Analyzer, Foss Tecator, Höganäs, Sweden). The crude protein (CP) content was calculated using the conversion factor of 6.25 (N × 6.25) [38].

#### 3.3.7. Fiber Content

The fiber content was determined according to the method of Van Soest *et al.* [41] as adapted for Komarec [42] and Senger *et al*. [43], using neutral detergent and digestion by autoclaving.

#### 3.3.8. Total Phenolic Content

The total phenolic content in the bee pollen extract was estimated following the Folin-Ciocalteau method, previously described by Moreira *et al.* [44]. Briefly, the reaction of 500 µL methanol extract (MeOH/bee pollen; 500 μL of 1:10 g∙mL^−1^), mixed with 500 µL of the Folin-Ciocalteau reagent and 500 µL of Na_2_CO_3_ (10% *w*/*v*) was kept in the dark at room temperature for 1 h. Then, the absorbance of the reaction mixture was read at 700 nm, using a Unicam Helios Alpha UV-visible spectrometer (Thermo Spectronic, Cambridge, UK). Galic acid standard solutions (0.01 × 10^−3^ to 0.08 × 10^−3^ M) were used for constructing the calibration curve. Total phenols content were expressed as mg of galic acid equivalents per g of bee pollen extract (GAEs).

#### 3.3.9. Flavonoid Content

For flavonoid contents determination, 250 μL of the methanol extracts of bee pollen were mixed with 1.25 mL of distilled H_2_O and 75 μL of a 5% NaNO_2_ solution. After 5 min, 150 μL of a 10% AlCl_3_⋅H_2_O solution were added. After 6 min, 500 μL of NaOH (1 M) and 275 μL of distilled H_2_O were added. The solution was mixed well and the intensity of color was measured at λ = 510 nm. Catechin standard solutions (0.022 × 10^−3^ to 0.34 × 10^−3^ M) were used for constructing the calibration curve. Total flavonoids content were expressed as mg of catechin equivalents per g of bee pollen (CAEs).

#### 3.3.10. Reducing Sugars

Reducing sugars were determined using the DNS (dinitrosalicylic acid) method [45].

### 3.4. Determination of the Fatty-Acid Profile

The crude fat (CF) was extracted with petroleum ether using an automatic Soxtec device (FOSS, Soxtec™ 2050, Höganäs, Sweden) [41 AOAC, 1995]. Fatty acid methyl esters were prepared from the extracted CF fraction by transesterification using MeOH in the presence of H_2_SO_4_. A sample containing 20 ± 0.5 mg of lipids was redissolved in 0.75 mL *n*-hexane; then 0.1 mL of 2 N KOH in MeOH was added and the solution was mixed in a vortex mixer, for 2 min (Model Reax 2000, Schwabach, Germany) and dried over anhydrous Na_2_SO_4_. After phase separation, the superior layer of *n*-hexane containing the fatty acid esters was removed and injected on a gas-chromatograph DANI model GC 1000 (Izasa, Barcelona, Spain) equipment, equipped with a split/splitless injector, a flame ionization detector (FID) and a Macherey-Nagel column (30 m × 0.32 mm ID × 0.25 µm: stationary phase of 50% cyanopropyl-methyl-50% phenylmethylpolysiloxane). The analysis of the methyl esters were performed using H_2_ as carrier gas at a pressure of 0.61 bar and at split ratio was of 1:40. The flow rate of the carrier gas was set at 4.0 mL∙min^−1^. A thermal gradient from 170 to 240 °C at 3.5 °C∙min^−1^ was used with the injector and detector temperatures at 240 °C. The injection volume was 1 µL. The Fatty acid methyl esters were identified by comparing the retention times of the peaks obtained in the bee pollen sample with those of known reference esters. The fatty acid composition was expressed as percent of major fatty acid methyl esters from the peak areas. Results were recorded and processed using Clarity 4.0.1.7 Software (DataApex, Podohradska, Czech Republic).

### 3.5. Statistical Analysis

All the experiments were conducted in a fully randomized manner. In a first approach, it was performed an overall descriptive analysis, by calculating means, standard deviations and relative standard deviation percentages (RSD (%)). To assess the linear dependence between pairs of variables, Pearson’s correlation coefficients were calculated and presented in the form of correlation matrix.

Shapiro-Wilk’s test was used to verify whether data follow normal distributions for each category of the independent variable and normal QQ plot was used to confirm if the variables lacking normality could have an approximately normal distribution. The homogeneity of variances was assessed using Levene’s test. Then, Anova one-way was applied when variables had groups with normality and homogeneity of variances and Kruskal-Wallis test, whenever there was homogeneity of variances (homocedastic variables) and no-normality. The Anova with Welch’s correction was applied in no-normality and no-homogeneity of variances (heterocedastic) variables.

To study the overall data’s variability, principal component analysis (PCA), an unsupervised multivariate technic, was used to generate a new set of orthogonal variables (linear combinations of the original variables), principal components (PCs), which represents a new subspace where the main variation present in the original data is mainly represented by the first components. It was used for visualization of hidden groups, possible outliers and, considering the projections of the original variables loadings, the variables’ influence in samples distribution.

All statistical studies [46,47] were performed using the open source statistical program R (version 2.15.1) [48] and adopting the 5% (p < 0.05) significance level.

## 4. Conclusions

The bee pollen is a natural product with a chemical composition that gives relevant biological properties, confirming its added value as a food product. In this study, for the first time, it was studied the overall physico-chemical characterization and fatty acid profile of pollen samples collected by the *Melipona mandacaia* (*Mandacaia* stingless bee) in two regions. Also, it was verified the presence of microorganisms in order to assess their food security, since the samples followed a normal processing either in all steps of pollen harvesting or at storage level. In this last study, the results showed that all samples were free of microbial contamination and only the presence of mesophilic aerobic microorganisms were detected, indicating that pollen manipulation was appropriate.

The physical and chemical parameters showed values within the stipulated by legislation, except for pH and moisture content, which samples had lower and higher values, respectively, than those established by Brazilian Legislation [15] or Argentine Food Code [14]. These two parameters results suggested that the storage conditions should be improved to avoid undesirable fermentations of this product. It should be noted that the phenolic compounds have a high content that contributes to an increase in antioxidant capacity of pollen although, a different situation was found for the total flavonoids content. Considering the natural variability within pollens’ samples results, it was possible to verify that samples’ geographic origin had a great impact in the physical and chemical characteristics, confirmed by PCA by showing that overall results had relevant information to distinguish partially samples between the two regions. This point was more evident when dealing with fatty acids profile, which was shown that data allowed complete distinction between samples from the two regions under study. The fatty acid profiles confirmed that the polyunsaturated compounds were significantly higher than the monounsaturated and saturated fatty acids. Hence, considering the ratio between unsaturated *vs.* saturated fatty acids, the pollen collected by *Mandacaia* stingless bee can be an asset as food product due to its nutritional quality.
